# Supplementary material for: Accelerated FRET-PAINT microscopy
Source: Mol Brain. 2018 Nov 22;11:70. doi: 10.1186/s13041-018-0414-3 (PMC6249777; doi:10.1186/s13041-018-0414-3)
Supplement: Supplementary file 1 — Accelerated FRET-PAINT Microscopy. Figure S1. Excitation spectra of Cy5 (black) and CF660R (red). Figure S2. A cross-sectional histogram of microtubules. Figure S3. Photo-induced damage of DNA probes. (DOCX 538 kb) [file 13041_2018_414_MOESM1_ESM.docx]

SUPPLEMENTARY FIGURES

**Accelerated FRET-PAINT Microscopy**

Jongjin Lee, Sangjun Park & Sungchul Hohng


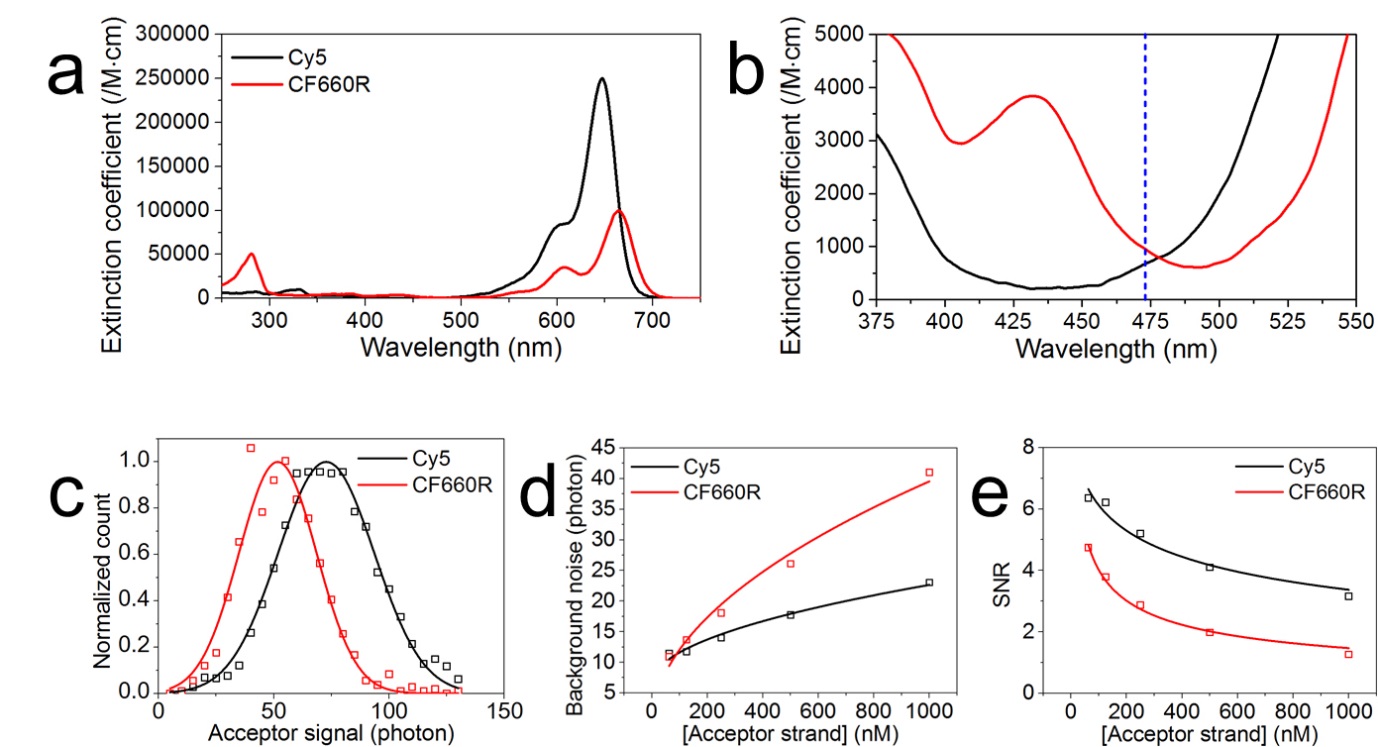


Figure S1 | Excitation spectra of Cy5 (black) and CF660R (red). (a) The overall spectra of Cy5 and CF660R. (b) A zoomed-in view of panel (a) around 473 nm. Excitation spectra of acceptor fluorophores were measured using a NanoDrop 2000 spectrophotometer (Thermo Fisher Scientific). The extinction coefficients of Cy5 and CF660R at 473 nm are 676 and 954/M·cm, respectively. (c) Acceptor signal of the CF488A-Cy5 (black) and CF488A-CF660R (red) pairs at 1.5 kW/cm^2^ excitation power recorded with an sCMOS camera and a band-pass filter. The signal is defined as the amplitude of a 2D Gaussian function of each single-molecule spot. Open squares indicate measured values and solid lines indicate fitted curves with a Gaussian function. The CF488A-Cy5 pair yields the higher intensity. (d) Background noise of the CF488A-Cy5 (black) and CF488A-CF660R (red) pairs with an sCMOS camera and a band-pass filter. The background noise is defined as the FWHM of a Gaussian function of the background signal. Open squares indicate measured values and solid lines indicate fitted curves with a square root of acceptor strand concentration. CF488A-Cy5 pair yields lower background noise. (e) SNR of the CF488A-Cy5 (black) and CF488A-CF660R (red) pairs recorded with an sCMOS camera and a band-pass filter. SNR is defined as the ratio of the signal to the background noise. Open squares indicate calculated values and solid lines indicate fitted curves with an inverse square root function of donor strand concentration. The CF488A-Cy5 pair yields the highest SNR at high acceptor strand concentration.

**
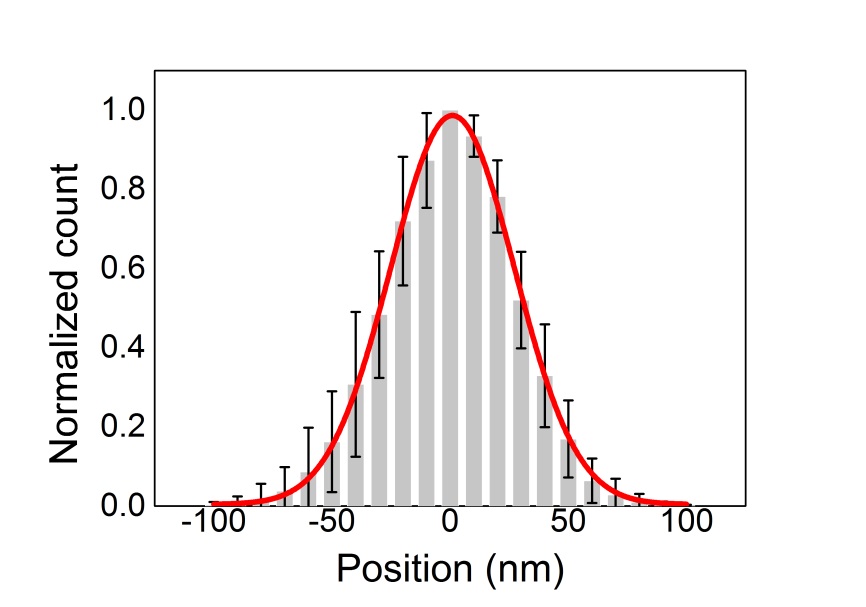
**

Figure S2 | A cross-sectional histogram of microtubules. 10 cross-sectional histograms of microtubules were obtained from Figure 3b. A bar graph indicates mean value and an error bar indicates standard deviation of 10 histograms. The bar graph was fitted to a Gaussian function and the corresponding FWHM was 61 nm.


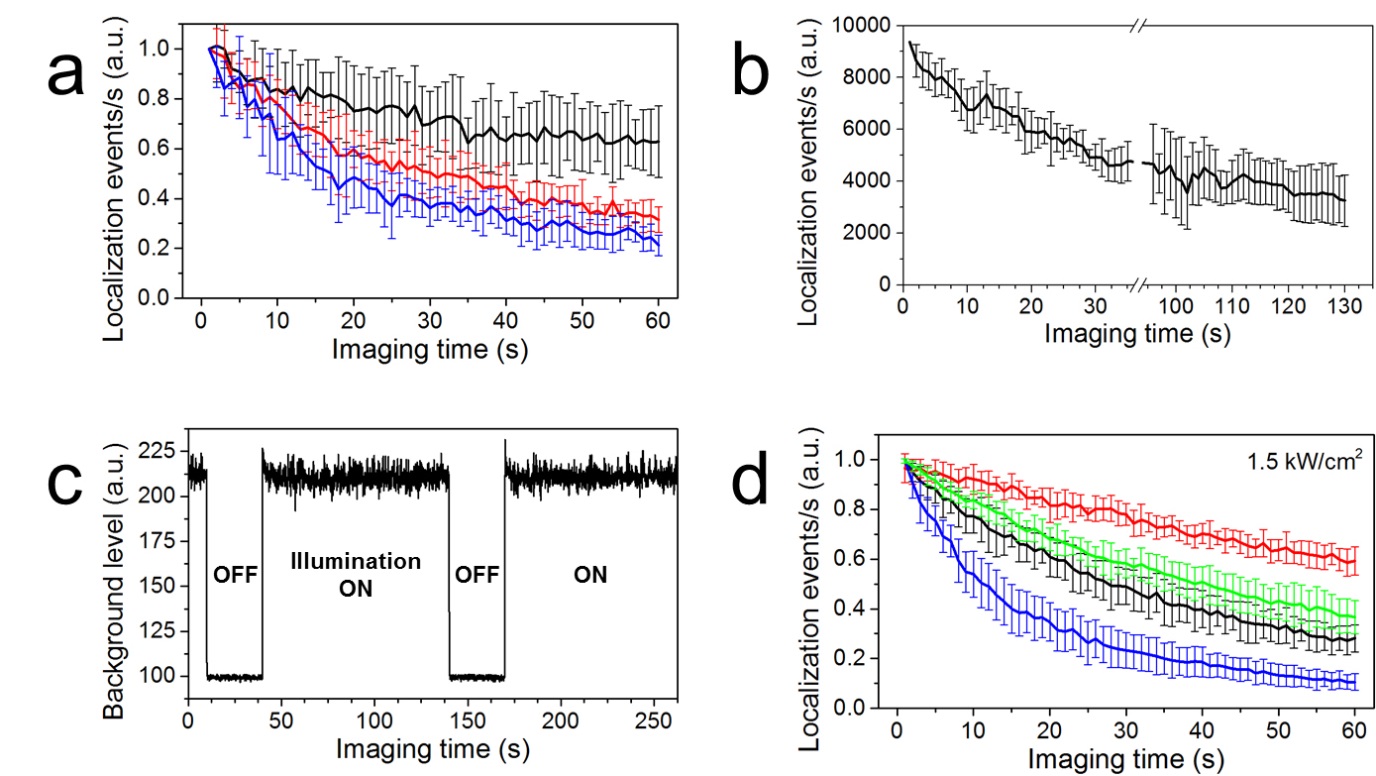


Figure S3 | Photo-induced damage of DNA probes. (a) The localization number per second was measured as a function of time at various illumination intensities (black, 1.5 kW/cm^2^; red, 4.5 kW/cm^2^; blue, 7.5 kW/cm^2^). The localization number was normalized to the initial value for each case. It is clear that the localization number decrease is more profound at higher illumination intensities. (b) To check whether this is a photobleaching effect or not, donor and acceptor strands were refreshed in the middle of the imaging. A COS-7 cell was imaged with 300 nM donor and acceptor strands in an imaging buffer for 35 s. And then the illumination was turned off. The sample chamber was refreshed thoroughly with the same imaging buffer with 300 nM donor and acceptor strands. The illumination was turned on at t = 95 s. And the same area was imaged for another 35 s. Because donor and acceptor strands were refreshed, the localization rate should be recovered to the initial value if the decrease was caused by photobleaching of donor or acceptor fluorophores. However, the localization rate was not recovered at all. Therefore, it is evident that the decrease of the localization number is not due to photobleaching. (c) As another way to check the photobleaching effect, the background noise level of donor strands was measured. The background noise level doesn’t decrease when the illumination is on. This result indicates that photobleaching is not the reason for the localization number decrease. (d) Localization rates were measured at the same illumination intensity with different samples. All samples were prepared with the same protocol but the decreasing rates are all different. This suggests that there may exist some steps which are critical to the photo-induced damage, but we could not efficiently control. Solid lines indicate average values and error bars indicate standard deviations. At least 5 cells were used for each cell preparation.
